# Lifestyle Modification in the Prevention and Management of Obesity

**DOI:** 10.1155/2016/5818601

**Published:** 2016-12-13

**Authors:** Shirley Telles, Bangalore N. Gangadhar, Kavita D. Chandwani

**Affiliations:** ^1^Patanjali Research Foundation, Haridwar, India; ^2^National Institute of Mental Health and Neurosciences, Bangalore, India; ^3^Department of Surgery, McGovern Medical School, University of Texas Health Science Center at Houston, Houston, TX, USA

Overweight/obesity is one of the most prevalent health problems on a global scale. This special issue intended to provide an academic platform for research which has used lifestyle interventions to facilitate and sustain weight loss. The published articles include a systematic review and original research articles.

In the review A. Baker et al. assessed the impact of long-term physical activity interventions for overweight/obese postmenopausal women, on indicators of obesity, physical capacity, and mental health measures. Five electronic databases (i.e., Medline, Cochrane Central, PubMed, PsycINFO, and Web of Science) between January 2005 and June 2015 were searched for relevant studies. After using appropriate search words and filters, 378 potentially relevant articles were found. For the final review ten articles of five RCTs were included. The findings showed that addressing intra- and interpersonal levels of behavior change resulted in several positive outcomes on adiposity measures (e.g., the BMI, fat mass, and measures of central obesity) and in physical capacity measures.

The original research included a study by S. J. Zizzi et al. which aimed to determine the reasons why people dropped out from a two-year, community-based weight management program and the consequences of the program. There were 400 participants of the community-based weight management program; their average length of participation was 9.8 months (SD 5.6). After applying rigorous methods of coding, seven main reasons emerged to explain why people dropped out of the program. The first three were (i) competing priorities (36.9 percent), medical reasons (22.8 percent), and negative experiences (13.1 percent). Both positive and negative consequences were noted after the program. The positive consequences included positive changes in attitude, behavioral changes, and physical benefits.

A study by B. Livia et al. assessed the motivation to adopt and sustain changes in lifestyle, with respect to two domains: (i) physical activity and (ii) nutrition. Motivation to change is considered a dynamic process with five stages. These are precontemplation; contemplation; determination; action; and maintenance. The participants were 100 overweight/obese persons (mean age 54.49, SD 11.03, 34 percent with comorbidity of type 2 DM). The intervention was given for 3 months, to groups of 5-6 patients with 26 sessions. After the intensive 3-month intervention the two stages of precontemplation and contemplation decreased significantly, whereas the two stages of action and maintenance increased significantly. This suggested that the intensive intervention (of 3 months) was adequate to initiate the process towards adopting and maintaining a healthier lifestyle.

Original research was carried out by C. Drenowatz et al. to compare physical activity patterns and changes in weight during weekdays and weekends. The study was conducted to examine differences in weekday and weekend physical activity along with associations between changes in physical activity patterns and changes in body weight over a one-year period. Participants were 338 healthy adults (age range 20–35 years; 53 percent male). Assessments were taken every 3 months for a year. The results emphasized the importance of physical activity during the weekend for long-term weight management.

The body image was studied in 663 university students in the Kingdom of Saudi Arabia by A. Khalaf et al. The participants' mean age was 20.4 (1.5) years. More than half of them had a normal body weight. An agreement between the actual, perceived, and ideal body image was found in 23 percent of the participants. Special behavioral, social, and economic factors correlated significantly with the desire either to be thinner or to be heavier. The study concluded that public health campaigns should target improving adolescents' body image as part of a strategy to avoid eating disorders.

An informal review by J. S. Puterbaugh discussed another aspect of obesity, i.e., the evolution of societal obesity from 1880 to 1980. The conclusion was that controlling societal obesity requires innovative analytic tools and an existential model which would include measures such as altering the built environment to encourage active lifestyles.

Hence this issue includes research which supports the importance of increased physical activity and correct nutrition at an individual level, as well as public health campaigns to address the physical and psychological aspects of obesity. This area of research requires further study as the importance of a lifestyle intervention in managing the growing problem of obesity appears reasonably convincing.

